# Clinical and Endoscopic Characteristics of Patients with Oligopolyposis

**DOI:** 10.3390/jcm14051562

**Published:** 2025-02-26

**Authors:** Ali Abu-Juma, Fahmi Abu-Galion, Zlata Lerner, Sarah Weissmann, Liza Ben-Shoshan, Waleed Alamour, Muhammad Abu-Arar, Naim Abu-Freha

**Affiliations:** 1 Institute of Gastroenterology and Hepatology, Soroka University Medical Center, P.O. Box 151, Beer-Sheva 84101, Israel; ali.abu.jumaa91@gmail.com (A.A.-J.); zlata.31@gmail.com (Z.L.); lizana1@clalit.org.il (L.B.-S.); 2 Internal Medicine Department, Soroka University Medical Center, P.O. Box 151, Beer-Sheva 84101, Israel; fahmiabu@gmail.com (F.A.-G.); dr.alamour@hotmail.com (W.A.); 3 Faculty of Health Sciences, Ben-Gurion University of the Negev, P.O. Box 653, Beer-Sheva 84105, Israel; sarahgrzebinski@gmail.com (S.W.); abumuh@clalit.org.il (M.A.-A.); 4 Emergency Department, Soroka University Medical Center, P.O. Box 151, Beer-Sheva 84101, Israel

**Keywords:** oligopolyposis, clinical features, outcomes, polyp, genetic testing, APC, MUTYH

## Abstract

**Background/Objectives**: Oligopolyposis is a rare condition characterized by 10 to 100 adenomas in the colon. We aimed to investigate the clinical and endoscopic features of patients with oligopolyposis by comparing patients who carried pathogenic mutations and those who did not. **Methods**: This retrospective study included patients with a cumulative count of 10–100 adenomas found in the colon, at a single center. Clinical, endoscopic, and genetic data were analyzed. **Results**: A total of 155 patients were identified as having oligopolyposis. Genetic testing using a multigene panel was performed among 85 (55%) patients, while founder or family mutation testing was performed among 7 (4.5%) patients. No genetic testing was carried out in 63 (40.5%) patients. Pathogenic polyposis-related mutations were identified in 14 (16%) out of 85 patients who underwent genetic testing. Among these, seven (50%) mutations were found in the *APC* gene and seven (50%) in the *MUTYH* gene. A significantly higher proportion of mutation carriers were of Arab ethnicity (35.7% vs. 4.2%, *p* < 0.001). There was no significant difference between carriers and non-carriers with regard to family history of polyps or cancer. Colorectal cancer was found to be the initial presentation in three (21%) carriers and five (7%) non-carriers. Colonic surgeries were reported among 4 (28.6%) carriers and 13 (18.6%) non-carriers. No significant differences in the rates of colorectal cancer or death were observed between carriers and non-carriers. **Conclusions**: Only a small proportion of patients with oligopolyposis were found to be mutation carriers, with significant ethnic differences in mutation frequency but no notable differences in clinical features, colorectal cancer rates, or mortality.

## 1. Introduction

Colonic adenomatous polyposis is a disease characterized by the formation of multiple adenomatous polyps in the colon that is well known to be a precursor for colorectal cancer (CRC). CRC is the third most common cancer, with a lifetime risk of about 4–5%, and the second leading cause of cancer mortality [1]. The adenoma–carcinoma sequence is a well-established pathway in CRC development, it is crucial to understand the natural history of adenomatous polyposis, particularly the less investigated polyposis syndrome, in people with 10–100 polyps [2,3].

Adenomas play a crucial role in the adenoma–carcinoma sequence, significantly contributing to the development of CRC and increasing the overall burden of the disease [4,5]. The most common sporadic polyps are adenomas, which develop without a genetic syndrome, and their prevalence is increasing with age [4,5]. Adenomas could be diagnosed during screening colonoscopy or colonoscopy for any indications [4,5]. Sporadic adenomas vary in size and histological features, and their potential malignant transformation is related to the adenoma’s size and high-grade dysplasia.

People with polyposis could have a genetic basis with germline mutations in polyposis-related genes, such as the *APC* gene, resulting in familial adenomatous polyposis (FAP), *MUTYH*-associated polyposis (MAP), or, rarely, in other genes such as *AXIN2, GREM1*, *NTHL1*, *POLE*, *POLD1*, or *MSH3* [6,7,8,9,10]. In a large cohort of 8676 individuals with polyposis, 24% of them had a pathogenic *APC* or *MUTYH* alteration. An increase in the number of adenomas increases the likelihood of having a mutation in the *APC* and *MUTYH* genes; however, most patients do not have a pathogenic genetic alteration [11].

Patients with oligopolyposis (10–100 polyps) should undergo genetic evaluation, with a focus on polyposis-related genes. In cases of no polyposis-related genetic mutation, patients are defined as having colonic adenomatous polyposis of unknown etiology (CPUE) [12]. A relative lack of understanding of CPUE hinders diagnosis and management. CPUE represents a unique clinical entity, often overshadowed by better-characterized syndromes like FAP and MAP, leading to a lack of focused research and clinical attention. There are few data published regarding this specific form of oligopolyposis, with only a small number of patients having been previously studied [13,14]. Unlike FAP or MAP, which have well-characterized genetic findings, CPUE lacks a clear genetic etiology, presenting challenges for surveillance and therapeutic decision making in patients with CPUE. In a previous retrospective study, which included 70 patients with CPUE, 69% of them were males and presented for genetic counseling at the age of 63.7 years, with 4 (5.7%) of them being diagnosed with CRC and 7 (10%) requiring colectomy [13]. Despite the clinical significance, CPUE remains largely absent from the medical literature, and only limited data are available regarding the prevalence, pathogenesis, natural history, and risk for colonic and extracolonic malignancies in patients with CPUE. The natural history, recommended follow-up, and surveillance of these patients have yet to be published, alongside their risk for colonic and extracolonic malignancies. Moreover, recommendations for the screening and surveillance of the colon, as well as other gastrointestinal parts and organs, are needed. Patients with CPUE may have unique risk profiles and clinical outcomes compared to other known polyposis syndromes, such as FAP or MAP, and need to be deeply investigated to avoid the delay of early interventions and decrease the risk of CRC. Moreover, exploration of the pathogenesis and genetic aspects of CPUE, including the environmental and epigenetic factors, is important for understanding the syndrome. All these points and aspects are crucial for clinicians’ daily decision-making process regarding these patients. The National Comprehensive Cancer Network (NCCN)’s recommendations for the management of patients with CPUE depend on the number of adenomas and the ability for endoscopic management: colonic surveillance with colonoscopy every 1 to 2 years is recommended for individuals with 20 or more colorectal adenomas, while those with 10–19 adenomas should be managed based on clinical judgment, with modification of surveillance frequency according to factors such as age and number of adenomas found during the latest colonoscopy [15]. In addition, the NCCN recommends baseline upper endoscopy at the time of the following colonoscopy surveillance by 20–25 years of age and repeat upper endoscopy according to the modified Spigelman score [15]. In general, these recommendations are derived from data on other hereditary polyposis syndromes, such as FAP and MAP, and their applicability to patients with CPUE remains unclear, necessitating further investigation. Moreover, screening and surveillance recommendations and guidelines are needed from various associations, encompassing diverse fields such as gastroenterology and genetics. CPUE management, especially extracolonic surveillance recommendations, remains a challenge [16].

This study aims to investigate the clinical endoscopic characteristics and risk factors for gastrointestinal cancer of patients with oligopolyposis, comparing those with a pathogenic mutation to those without a mutation (CPUE). This study aims to address part of the known gaps in our knowledge of CPUE. By addressing these gaps, this research seeks to enhance our understanding of CPUE.

## 2. Materials and Methods

We conducted a retrospective study that included patients with oligopolyposis with a count of 10–100 adenomas either during a single colonoscopy or cumulatively, over multiple procedures. This study was performed in a single-center setting, at a tertiary university Medical Center, which serves as a regional referral center for complex gastrointestinal cases. In general, patients who are diagnosed with oligopolyposis are referred to the high-risk patient clinic in the gastroenterology department or to the genetics institute for further investigation. The patients continue follow-up and surveillance in the gastroenterology department.

### 2.1. Study Population

The study population included patients with oligopolyposis who had been identified by the high-risk outpatient clinic of the gastroenterology and hepatology department at Soroka University Medical Center, a tertiary academic referral center, in Southern Israel, which provides a tertiary service for about 700,000 people. Patients 18 years old or older who had at least 10 adenomas and less than 100 adenomas were enrolled in this study and included in the analysis. Patients were excluded if they had fewer than 10 adenomas and other types of polyps (hyperplastic, hamartomatous polyps, or other non-adenoma polyps); adenomas were confirmed through histopathological examination according to the data collected. The dataset included all patients meeting the diagnostic criteria for oligopolyposis. The analysis was further stratified to compare the subset of patients who underwent genetic investigation. We presented the results of all patients who had oligopolyposis and then focused on those who underwent genetic investigation. The study population was divided into two subgroups based on the genetic testing results: (1) patients with confirmed polyposis-related mutations and (2) those without identifiable mutations.

### 2.2. Data Collection

Clinical, genetic, and endoscopic data of the included patients were collected. The data were collected from computerized medical files including the patients’ endoscopy reports. Demographics (age, gender, and ethnicity), smoking status, family history of polyps, and family history of CRC were collected. Data from endoscopies, including colonoscopies and esophagogastroduodenoscopies (EGDs), were also retrieved. In addition, the histopathology reports were reviewed, and the adenomas’ histopathology was confirmed. Detailed information about the adenomas, including first presentation, genetic investigation, family history, and cumulative polyp count, was collected. Additionally, data about colonic cancer and other extracolonic malignancies, colonic surgery, and mortality were also collected. The demographics, clinical, endoscopic, family history of CRC (first or second degree) or polyps, and outcomes were compared between the study groups, split between those with polyposis-related carrier mutations and those with oligopolyps but without genetic mutations in a polyposis-related gene (CPUE).

### 2.3. Statistical Analysis

Patient characteristics were presented as the mean ± standard deviation (SD) for continuous variables and percentages (%) for categorical variables. Categorical variables were compared using the chi-square test. Continuous variables were examined with Student’s t-test. Logistic regression models were used to examine the univariate relationships between various risk factors and the odds of having a polyposis-related genetic mutation, allowing for the identification of potential predictors and trends. No multivariable logistic regression was performed due to the lack of significance of most variables included in our analysis.

All statistical analyses were performed using IBM SPSS version 29 (Chicago, IL, USA). *p*-values less than 0.05 were considered statistically significant. The study protocol was approved by the Institutional Helsinki Committee of Soroka University Medical Center, with approval number 0099-24-SOR. To ensure ethical compliance, informed consent was waived due to the retrospective design, but patient confidentiality was maintained throughout the data collection and analysis process.

## 3. Results

**Figure 1:**
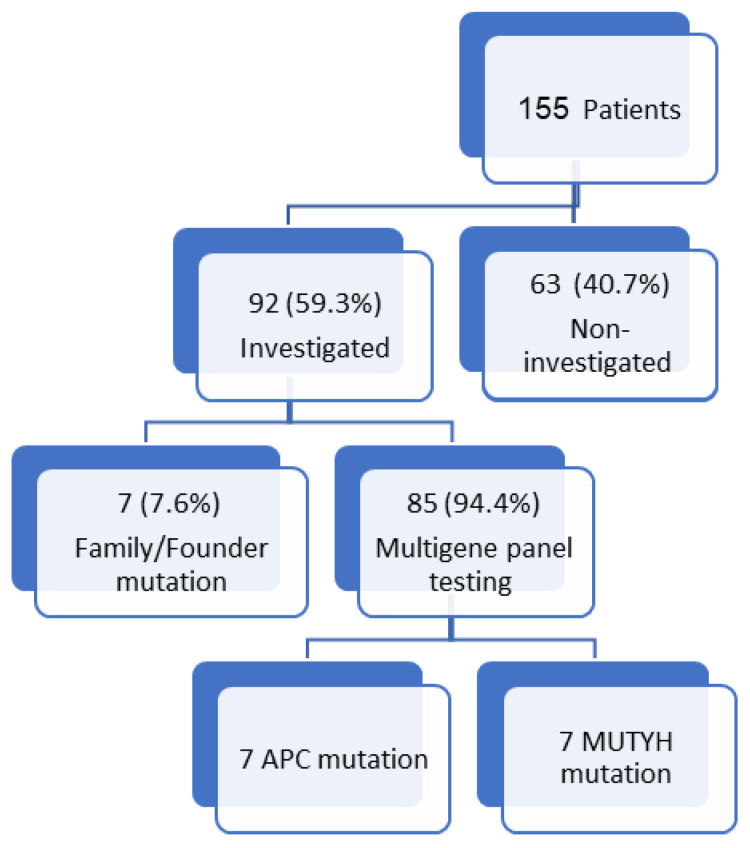
Flowchart of the study’s subjects.

The flowchart of patients included in this study is shown in Figure 1.

The baseline characteristics of patients with oligopolyposis (10–100 adenomas) are summarized in Table 1. One hundred and fifty-five patients between the years 2014 and 2024 were included as they had more than 10 adenomas. A total of 92 (59.3%) of the patients underwent genetic investigation, with 85 (94.4%) of them undergoing multigene panel testing and family/founder mutation being performed in 7 (7.6%) patients. The mean age was 68.6 ± 12.4 years, with 96 (61.9%) males, and 13 (8.4%) patients of Arab Bedouin ethnicity. A total of 66 (42.6%) of the patients were smokers, and the mean BMI was 30.2 ± 3. Only 16 (10.3%) of the patients had a family history of polyps, and 26 (16.7%) of the patients had a family history of CRC. Regarding the polyp count, 67 (43.2%) had 11–20 polyps, and 53 (34.2%) had ≥31 polyps, while 13 (8.4%) of the patients had 10 polyps, and 22 (14.2%) had 21–30 polyps. In most of the patients, 141 (91%) of the total, a diagnosis of colonic polyps was the first presentation, while 14 (9%) of them had CRC as the first presentation. Sixteen (10.3%) of the patients were diagnosed with CRC during their lifetime either during the study period or follow-up.

### 3.1. Polyposis-Related Mutation Carriers

Genetic testing with multigene panel testing (MGPT) was performed in 85 patients, and pathogenic polyposis-related mutations were found in 14 (16%) of the 85 patients who underwent genetic investigation. Seven (50%) mutations were found in the *APC* gene, and seven (50%) were found in the *MUTYH* gene. The comparison between the polyposis-related mutation carriers and those without mutation (CPUE) is summarized in Table 2. Comparison of the mutation carriers to the non-carriers revealed no significant differences between them in terms of age and gender (64.3 ± 15.6 years vs. 67.1 ± 10.3 years, *p* = 0.401, 57.1% females vs. 32.4% females, *p* = 0.079). However, a significantly higher rate of patients of Arab Bedouin ethnicity was found among the carriers (35.7% vs. 4.2%, *p* < 0.001). No significant difference was found regarding the family history of polyps and family history of CRC (28.6% vs. 15.5%, *p* = 0.241, and 14.3% vs. 7%, *p* = 0.368, respectively). Colorectal cancer was found to be the first presentation among three (21%) carriers and five (7%) non-carriers. In addition, no significant difference between the groups was found regarding colonic surgery (four patients (28.6%) vs. thirteen patients (18.6%), *p* = 0.395), CRC rate (three cases (21.4%) vs. seven cases (9.9%), *p* = 0.219), and mortality rate (zero vs. five cases (7%), *p* = 0.306). Among the four mutation carriers who underwent colonic surgery, three (75%) had undergone surgery due to CRC, while one patient underwent surgery due to a high polyp burden. Among the thirteen non-carriers who underwent colonic surgery, seven (54%) had undergone surgery for CRC, while six underwent surgery due to a high polyp burden. The highest prevalence of polyposis-related mutations was found among patients with 31 adenomas and more: nine (64.3%) mutation carriers were in this group. Meanwhile, the lowest prevalence was among patients with 10 polyps, with one patient (7.1%) among these having a polyposis-related mutation. The characteristics of the 14 mutation carriers are shown in Table 3, providing a comprehensive overview of their demographic, clinical presentation, and genetic profiles. The data include age, gender, ethnicity, gene, family history of polyps and CRC, and cumulative number of polyps. Additionally, data on colonic surgery, CRC, and extracolonic cancer are summarized in Table 3. The age span at diagnosis was between the ages 27 and 73 years.

Four (28.6%) of the patients underwent surgery, and none of the carriers were diagnosed with extracolonic malignancies.

### 3.2. Univariate Analysis for Carrying Polyposis-Related Mutations

In the univariate analysis for the risk of carrying a polyposis-related mutation, a Bedouin Arab ethnicity was found to be a predictor for carrying a pathogenic mutation in the MGPT, with an odds ratio of 12.593 (95% CI, 2.565–61.829, *p* = 0.002), while other factors, such as sex, FHCRC, family history of polyps, and the count of polyps, were not found to be risk factors for carrying a mutation. The univariate analysis is presented in Table 4. Since the predictors (other than ethnicity) were not statistically significant, no multivariate analysis was performed.

## 4. Discussion

Oligopolyposis is a condition that requires further evidence-based research to understand the prevalence of colonic and extracolonic malignancies and recommend surveillance among those affected with the disease [12,16].

In this study, we present several important findings: (1) Only part of the patients with oligopolyposis underwent a genetic investigation for oligopolyposis. (2) A total of 16.5% of the patients who underwent MGPT were found to carry a polyposis-related mutation. (3) The CRC rate among mutation carriers was 21.4% and 9.9% among patients with CPUE. (4) None of the study participants underwent genetic investigation, whether in the mutation carrier group or the group with CPUE. (5) A total of 35.7% of the mutation carriers were of Arab Bedouin origin compared to 4.2% of the patients with CPUE.

Only 55% of the patients with oligopolyposis diagnosed in the endoscopy department underwent multigene panel testing. Oligopolyposis appears to be a relatively common condition among patients with CRC. In a previous study analyzing 1573 colectomy specimens of patients with CRC, oligopolyposis was observed in 9.47% of specimens [17]. The presence of ≥10 adenomas upon fecal immunochemical test (FIT)-based screening was found in 2.2% of the patients, with a cumulative incidence of advanced neoplasia in 16% [18,19]. These findings highlight the need for increasing awareness among gastroenterologists and surgeons regarding the importance of further genetic investigation of patients with oligopolyposis. Genetic counseling integration with routine referrals to genetic counselors should be part of oligopolyposis investigations.

For patients presenting with more than 10 cumulative adenomatous polyps, genetic evaluation is crucial to identify potential hereditary polyposis syndromes and those with specific syndromes, such as FAP, AFAP, or MAP. The early identification of these conditions is important as it enables tailored surveillance and management strategies. Patients with oligopolyposis need to be genetically evaluated via comprehensive genetic testing, including analyses of the *APC* gene, *MUTYH* gene, and other polyposis-related genes, such as *POLE* and *POLD1*. The NCCN recommends multigene panel testing of all polyposis and CRC genes among patients with polyposis of 10 adenomas or more [15].

In our study, 16.5% of the patients who underwent MGPT were found to carry a polyposis-related mutation. This prevalence was lower than that found in a previously reported study, where *APC* and *MUTYH* mutations were identified in 24% of patients [11]. One report investigating a cohort of 3197 patients with adenoma found a much lower prevalence of adenomatous polyposis mutations (8.1%), subdivided between patients with 10–19 colonic adenomas (2.3% had a mutation), patients with 20–99 adenomas (8.5%), and patients with 100 or more adenomas (41.3%) [20]. The variability in prevalence in different studies could be attributed to different reasons, such as the selection criteria of the patients, population-specific genetic backgrounds, and differences in genetic testing. Notably, the prevalence of pathogenic mutations tends to increase with the adenoma burden, underscoring the importance of the adenoma count as a key factor in genetic evaluation. Moreover, advancements in genetic testing technology, including next-generation sequencing (NGS), have enabled the detection of pathogenic variants not only in known and common genes, such as *APC* and *MUTYH*, but also in additional genes associated with polyposis syndromes, such as *POLE, POLD1,* and *NTHL1*, improving diagnostic accuracy. The clinical phenotypes of these pathogenic mutations vary greatly depending on their genetic characteristics [21]. Comprehensive genetic testing and standardization of the criteria for genetic testing in patients with multiple adenomas will help assess the real prevalence of mutations among patients with polyposis.

The CRC rate was 10.3% (16 patients) among all the patients with oligopolyposis included in our study, with a higher rate among mutation carriers, accounting for 21.4% (three cases) and a lower rate of 9.9% (seven cases) among patients with CPUE. This finding emphasizes the increased risk of CRC among patients with oligopolyposis, particularly those with a genetic mutation. The rate of CRC was higher in our cohort compared to the previously reported rate of 5.7% among patients with CPUE [13]. Still, in the above study, other patients were diagnosed with advanced neoplasia, including three cases of intramucosal cancer and cases of a malignant polyp, resulting in a rate of 11%, which is comparable to our result [13]. These findings underscore the importance of the identification and colonic surveillance of patients with oligopolyposis because of their increased risk for CRC. Indeed, an increased risk for CRC was found among both patients with genetic mutations or those without a mutation (CPUE). The incidence of CRC in patients with oligopolyposis is an important issue, especially in relation to early detection and providing curative treatment. However, the data in the literature regarding the risk of CRC are scant, particularly among patients with CPUE. Additional future studies involving a large number of patients are essential to assess the frequency rate of CRC among this specific group of patients. Knowledge regarding the risk of CRC and other extracolonic malignancies is critical because of its significance for the surveillance interval. Moreover, understanding the impact of risk factors for CRC on patients with oligopolyposis (such as adenoma burden, histopathological type of adenoma, adenoma size, other risk factor, specific genes involved, and type of mutation) could play a role in advancing personalized medicine and optimizing colonic surveillance strategies for each individual.

Regarding extracolonic malignancies, none of the study participants who underwent genetic investigation, whether in the mutation carrier group or the CPUE group, were diagnosed with an extracolonic malignancy. However, previous studies have reported the occurrence of extracolonic malignancies at varying rates, including gastroduodenal neoplasia and breast, lung, or other malignancies [13,14]. Even studies with small sample sizes comparable to ours showed extracolonic malignancies [13,14]. This finding could be explained by different ethnic backgrounds of the patients, with different genetic predispositions and environmental exposures influencing the risk for extracolonic malignancies. Moreover, the study design and varying follow-up periods may affect the findings of different studies.

In the comparison between the mutation carriers and non-carriers (CPUE), no significant differences were found regarding age, family history of polyps or CRC, and death. While a higher prevalence of CRC as the first presentation was observed among the mutation carriers, the difference was still not statistically significant. A previous report with a similar sample size to ours detected a much higher frequency rate of CRC, with 35% among mutation carriers and non-mutation carriers, compared to only 11.7% of the genetically investigated patients in our study [22]. Another study with a large cohort of patients with adenoma reported a CRC rate of 18.8% [20]. According to findings from upper endoscopies, no polyps or malignancies were diagnosed among mutation carriers, while one patient had gastric polyps in the CPUE group. This may indicate that the risk for gastric and duodenal polyps and malignancies is very low. However, another previous study, which included 120 patients, showed that 7% (9 patients) were diagnosed with gastroduodenal neoplasia [14]. This discrepancy may be attributable to differences in the study populations, diagnostic criteria, follow-up periods, or inclusion criteria.

This study demonstrated a significant difference in ethnicity between carriers and non-carriers. A total of 35.7% of the mutation carriers were of Arab Bedouin origin compared to 4.2% of the patients with CPUE. Additionally, in the multivariate analysis, ethnicity was a significant predictor for mutation carrier status. Differences between ethnicities could be a result of high consanguinity and unique genetic features [23,24], underscoring the potential role of not only the genetic background but also the sociocultural factors unique to specific populations. This aspect of ethnicity remains underexplored in the literature on patients with CPUE or oligopolyposis. However, ethnicity may have an impact on the frequency rates of mutations and the risk for CRC or other cancers. A previous study with a large number of patients with polyposis with 10 or more polyps, (3197 patients), subdivided the sample according to ethnicity, with 72.1% of Caucasian ethnicity and 2.7% of Asian origin [20]. In our study context, patients of Arab Bedouin origin are a minority of the Arab population in Israel, characterized by a high rate of consanguinity and a unique sociocultural context, shaped by rapid modernization and urbanization processes. These findings highlight the need for the special consideration and investigation of distinct populations and ethnicities, as well as further research on specific populations, for assessing the frequency rate of mutations and the risk of CRC or other cancers. Up until now, ethnicity as a risk factor has not been considered in the surveillance interval for post-polypectomy surveillance in the various guidelines [25]; additional studies are required to investigate this issue further, particularly discussions on whether patients from different ethnic groups may benefit from tailored surveillance recommendations or different criteria for genetic testing or specific genetic investigation such as next-generation testing or targeted testing for founder mutations.

A family history of CRC or polyps is a risk factor for CRC or polyps; however, in our cohort of patients with oligopolyposis, only a small percentage had a family history of CRC (16.7%) or polyps (10.3%). Among patients with CPUE in another study, 22.1% of the patients had a family history of CRC compared to 15.5% of the patients in our study, and 31.4% of the patients vs. 11.4% of the patients in our study had a family history of polyps [13]. The rate of family history of CRC or polyps was relatively low in our study and other studies. Most patients with oligopolyposis do not have a family history of polyps; this could be explained by de novo mutations among mutation carriers and patients with CPUE who are the first to be affected in their family. These findings underscore the importance of comprehensive genetic testing and screening of family members of the index patient to rule out polyposis among additional family members.

In summary, colonic oligopolyposis is of unique interest, particularly CPUE, but limited data are available, with only small cohorts having previously been studied. Our research contributes important insights regarding colonic oligopolyposis. There are notable differences between our study and previous studies in terms of mutation prevalence, CRC incidence, family history of CRC, and family history of polyps. Ethnicity appears to play a role in the prevalence of polyposis-related mutations among patients with oligopolyposis. While there are established guidelines for the surveillance of patients with sporadic polyps and those with syndromes such as familial adenomatous polyposis (FAP) and *MUTYH*-associated polyposis (MAP) [26], large studies are needed to provide more evidence-based recommendations for patients with CPUE. Further research will help us understand and clarify important information, such as the risk for CRC, and inform guidelines on surveillance intervals for CRC and extracolonic cancers, which were not common in our subanalysis. Due to the lack of large studies, the establishment of a prospective database or international consortium for patients with CPUE could be highly beneficial. Such an international consortium for CPUE, with cases from different countries, would enable the collection of standardized data from diverse populations, including different ethnic groups, addressing current gaps in knowledge. Such an initiative could significantly enhance our knowledge of this topic and affect clinical practice. This is evident from previous databases, such as that for Lynch syndrome, the Prospective Lynch Syndrome Database (PLSD), which provided valuable insight into Lynch syndrome and impacted clinical decision making and management strategies.

Further research is needed with a large number of patients, investigating the role of ethnicity and the risk for extracolonic cancer, which will help establish evidence-based guidelines for the surveillance of colonic and extracolonic cancers. In addition, future research should integrate not only genetic investigations but also multi-factor analyses, incorporating clinical, environmental, dietary, and microbiome-related factors. All these factors could have an impact on the development of polyps, the burden of the polyps, and the risk for colonic or extracolonic cancers.

The clinical implications of the present study are, first of all, the importance of referring patients with oligopolyposis for genetic investigation and the early identification of hereditary polyposis syndrome. In addition, polyposis-tailored genetic panels are important for genetic investigation, and special consideration is needed for specific ethnic groups via targeted genetic testing.

The limitations of the present study are the retrospective, single-center design and the lack of genetic investigation in some of the patients. In addition, the patients in our study were of Jewish and Arab Bedouin origin, so our findings may not fully represent the broader population of patients with oligopolyposis or CPUE. The incomplete genetic testing coverage of our cohort is another limitation, with 40.5% of patients remaining untested; this created a potential bias, as untested patients may have harbored undetected pathogenic mutations, possibly leading to the underestimation of the true prevalence of oligopolyposis-related genetic variants, or they could have had a different clinical phenotype.

## 5. Conclusions

A relatively small proportion of patients with oligopolyposis were found to be mutation carriers, with significant ethnic differences in the mutation frequency rate but no significant differences in the clinical features, colorectal cancer rate, or death. This study highlights that ethnic and genetic factors influence the prevalence of CRC among patients with oligopolyposis. Performing multigene panel testing is essential for differentiating between mutation carriers and patients with CPUE, particularly among specific ethnic groups with a high rate of consanguinity or genetic disease.

### 5.1. Current and Next Challenges, as Well as Future Perspectives

#### 5.1.1. Challenges Related to CPUE

One of the main challenges in CPUE is the limited access to genetic testing, with only a portion of patients undergoing genetic evaluation and even those tested often receiving only a partial genetic investigation. Additionally, the lack of data on specific uninvestigated ethnic populations limits the genetic assessment. Another significant challenge is the absence of well-defined surveillance guidelines. Due to the lack of large-scale studies involving a substantial number of patients with CPUE, it remains difficult to establish clear, evidence-based recommendations for the surveillance of colonic and non-colonic cancers.

Furthermore, decision making regarding prophylactic surgery versus continued endoscopic surveillance remains complex and highly individualized, as there are no universally accepted protocols to guide clinicians and patients in optimally managing the disease.

#### 5.1.2. Future Perspectives

Expanding genetic testing for patients with oligopolyposis is essential. This includes incorporating whole-genome sequencing in clinical studies to identify potential polygenic risk factors, additional pathogenic mutations, and novel genetic markers, beyond *APC* and *MUTYH*.

Ethnic-specific genetic studies are particularly important due to the observed differences in mutation frequency rates among various populations. Understanding these disparities could help us develop tailored screening and management strategies.

Exploring targeted therapies and chemoprevention strategies for patients with oligopolyposis, especially those with known genetic mutations, could significantly improve patient outcomes and reduce disease progression. Finally, conducting large-scale, multi-center, and multinational studies, alongside establishing a comprehensive database for long-term clinical outcomes, is important. Such collaborative efforts will help address existing gaps in knowledge and support the development of standardized guidelines for patients’ genetic investigation and surveillance of malignancies.

## Figures and Tables

**Table 1:** Characteristics of cohort with colonic adenomatous polyposis of unknown etiology, n (%).

Patients with Oligopolyposis (155)	
Age, mean ± SD	68.6 ± 12.4
Sex, male	96 (61.9%)
Ethnicity	
Jewish	142 (91.6)
Arab	13 (8.4)
Smoking	66 (42.6)
Body mass index (BMI)	30.2 ± 3
Genetic investigation	
Multigene panel testing	85 (55)
Founder/family mutation	7 (4.5)
No investigation	63 (40.5)
Family history of polyps	16 (10.3)
Family history of colorectal cancer	26 (16.7)
Cumulative polyps	
10 polyps	13 (8.4)
11–20 polyps	67 (43.2)
21–30 polyps	22 (14.2)
≥31 polyps	53 (34.2)
First presentation	
Polyps	141 (91)
CRC	14 (9)
CRC anytime	16 (10.3)
Extracolonic malignancies	5 (3.2)

**Table 2:** Polyposis-related mutation carries vs. non-carrier patients.

	Mutation Carrier n = 14	Non-Carriers n = 71	*p*-Value
Age, mean ± SD year	64.3 ± 15.6	67.1 ± 10.3	0.401
Gender, female	8 (57.1)	23 (32.4)	0.079
Ethnicity, Arab	5 (35.7)	3 (4.2)	<0.001
Smoking	7 (50)	40 (58)	0.583
Family history of polyps	2 (14.3)	8 (11.4)	0.763
Family history of CRC	4 (28.6)	11 (15.5)	0.241
Colonic surgery	4 (28.6)	13 (18.6)	0.395
First presentation			
Polyps	11 (78.6)	66 (93)	
CRC	3 (21.4)	5 (7)	0.092
Cumulative polyps			0.328
10 polyps	1 (7.1)	3 (4.2)	
11–20 polyps	2 (14.3)	25 (35.2)	
21–30 polyps	2 (14.3)	14 (19.7)	
≥31 polyps	9 (64.3)	29 (40.8)	
Extracolonic cancers	0	0	
CRC any time	3 (21.4)	7 (9.9)	0.219
Death	0	5 (7)	0.306

**Table 3:** Characteristics of polyposis-related mutation carriers.

	Age *	Gender	Ethnicity	First Presentation	Gene	Family History of CRC	Family History of Polyp	Cumulative Polyp Count	Surgery	Other Cancers
1	66	Male	Jewish	CRC +2 adenoma	*APC*	No	No	74	Yes	No
2	27	Female	Bedouin	30 adenomas	*ACP*	No	No	70	No	No
3	49	Female	Jewish	7 adenomas	*APC*	Yes	No	45	No	No
4	54	Male	Bedouin	13 adenomas	*APC*	Yes	Yes	35	Yes	No
5	64	Female	Jewish	8 adenomas	*APC*	Yes	No	27	No	No
6	68	Male	Jewish	15 adenomas	*APC*	No	No	27	No	No
7	60	Male	Jewish	1 adenoma	*APC*	No	No	13	No	No
8	42	Female	Bedouin	25 adenomas	*MUTYH*	No	No	100	No	No
9	58	Female	Bedouin	10 adenomas	*MUTYH*	No	No	61	Yes	No
10	43	Male	Bedouin	CRC	*MUTYH*	No	No	60	Yes	No
11	60	Female	Jewish	1 adenoma	*MUTYH*	Yes	No	48	No	No
12	73	Male	Jewish	6 adenomas	*MUTYH*	No	No	45	No	No
13	60	Female	Jewish	8 adenomas	*MUTYH*	No	Yes	15	No	No
14	48	Female	Jewish	CRC	*MUTYH*	No	No	10	No	No

* Age at presentation.

**Table 4:** Univariate analysis for polyposis-related mutations.

	Univariate Analysis for Carrying a Polyposis Mutation		
	OR	95% CI	*p*-Value
Age	0.980	0.934–1.027	0.399
Sex, female	2.783	0.864–8.960	0.086
Ethnicity, Bedouin	12.593	2.565–61.829	0.002
FHCRC	2.182	0.579–8.215	0.249
Family history of polyps	1.292	0.244–6.849	0.764
10 polyps	1.744	0.168–18.094	0.641
11–20 polyps	0.307	0.064–1.480	0.141
21–30 polyps	0.679	0.136–3.385	0.636
≥31 polyps	2.607	0.792–8.580	0.115

## Data Availability

No additional data are available.

## Abbreviations

The following abbreviations are used in this manuscript:

CPUE	Colonic adenomatous polyposis of unknown etiology
CRC	Colorectal cancer
FAP	Familial adenomatous polyposis
EGD	Esophagogastroduodenoscopy
